# Non-Stacked γ-Fe_2_O_3_/C@TiO_2_ Double-Layer Hollow Nanoparticles for Enhanced Photocatalytic Applications under Visible Light

**DOI:** 10.3390/nano12020201

**Published:** 2022-01-07

**Authors:** Xun Sun, Xiao Yan, Huijuan Su, Libo Sun, Lijun Zhao, Junjie Shi, Zifan Wang, Jianrui Niu, Hengli Qian, Erhong Duan

**Affiliations:** 1 Yantai Key Laboratory of Gold Catalysis and Engineering, Shandong Applied Research Center of Gold Nanotechnology (Au-SDARC), School of Chemistry & Chemical Engineering, Yantai University, Yantai 264005, China; yhzheng@ytu.edu.cn (X.Y.); suhuijuan2012@ytu.edu.cn (H.S.); sunlibo@ytu.edu.cn (L.S.); zhaoljytu@ytu.edu.cn (L.Z.); junjieshi@ufl.edu (J.S.); 2 Biomedical Research Center, Northwest Minzu University, Lanzhou 730000, China; 3 School of Environmental Science and Engineering, Hebei University of Science and Technology, Shijiazhuang 050018, China; niujianrui@hebust.edu.cn (J.N.); 211706022@stu.hubest.edu.cn (H.Q.)

**Keywords:** double-layer hollow nanoparticles, non-stacked structure, synergistic effect, phenol, photodegradation

## Abstract

Herein, a non-stacked γ-Fe_2_O_3_/C@TiO_2_ double-layer hollow nano photocatalyst has been developed with ultrathin nanosheets-assembled double shells for photodegradation phenol. High catalytic performance was found that the phenol could be completely degraded in 135 min under visible light, due to the moderate band edge position (VB at 0.59 eV and CB at −0.66 eV) of the non-stacked γ-Fe_2_O_3_/C@TiO_2_, which can expand the excitation wavelength range into the visible light region and produce a high concentration of free radicals (such as ·OH, ·O^2−^, holes). Furthermore, the interior of the hollow composite γ-Fe_2_O_3_ is responsible for charge generation, and the carbon matrix facilitates charge transfer to the external TiO_2_ shell. This overlap improved the selection/utilization efficiency, while the unique non-stacked double-layered structure inhibited initial charge recombination over the photocatalysts. This work provides new approaches for photocatalytic applications with γ-Fe_2_O_3_/C-based materials.

## 1. Introduction

Phenol containing wastewater is produced in many industries, such as pharmaceuticals, polymer, dye, etc. [1]. Once phenol is released into the environment, it cannot be degraded inherently under the natural environment, and it causes harm to human health [2,3]. The phenol degradation is complex due to the high conjugated molecular system [4] and lower phenol concentration is also toxic [2]. Therefore, designing an environmentally friendly, cost-effective and highly efficient approach for phenol treatment is urgent. Photocatalysis is a simple technology among water treatment technologies [5,6,7], owing to its high mineralization and sturdy treatment efficiency [8,9].

Titanium dioxide (TiO_2_) is the most-used semiconductor material in the photocatalytic field, due to its chemical/physical excellent profound mineralization capability and stability under ultraviolet light [10]. However, two significant problems need to be solved: The low specific surface area of TiO_2_ (50 m^2^/g for P25, TiO_2_ with a mixed rutile phase, and anatase phase with an average particle size of 25 nm) confines its adsorption capacity for pollutant molecules. Additionally, it has a wide energy gap and is challenging to utilize visible light. Therefore, the preparation of a TiO_2_ catalyst with an extensive specific surface area and response to visible light is the bottleneck problem in applying TiO_2_.

To enhance the visible light absorption capacity, the commonly used means [11] are constructing heterojunction, defect engineering, and element doping. The construction of heterojunction catalysts can enhance the absorption of visible light and enhance the separation efficiency of photogenerated carriers because photogenerated electrons and holes can migrate from one phase to another through the two-phase interface, which can effectively inhibit the recombination of the photogenerated carriers [12,13,14]. More recently, double-layer hollow microsphere semiconductor catalysts have been applied as photocatalysts [15]. By virtue of the advantages of the double-layer structure, the heterogeneous junction catalyst based on the double layer structure is explored.

The construction of a core-shell heterojunction, γ-Fe_2_O_3_, maybe an ideal inner material that can improve solar energy utilization efficiency, which can also simplify the separation process with magnetic properties [16]. Most of the related work has concentrated on the design of functional cores or shells, but the effect of space accumulation between the two layers has been neglected. Based on the photocatalysis mechanism [17,18,19], the specific energy barrier formed at the interface between the core and the shell can be changed by adjusting the overlap mode between the two layers, promoting the separation of photogenerated carriers. Hence, the structure of the outer TiO_2_ needs a directional design. It is an urgent problem to build a non-simple stacked TiO_2_ shell and increase its specific surface area.

Recently, Zhao’s group [20] developed a TiO_2_ shell material composed of ultrathin nanosheets. This shell material composed of ultrathin TiO_2_ nanosheets increases the specific surface area of the catalyst. It constructs a non-simple stacked core-shell structure, making the catalyst appear in a porous state, which improves the light utilization rate and the adsorption capacity of pollutant molecules. Because the structure permits light scattering and refraction, the diffusion distance of photogenerated electron-hole pairs is reduced, and the light utilization is maximized [4].

This study demonstrates a facile route to synthesize non-stacked γ-Fe_2_O_3_/C@TiO_2_ double-layer hollow nanoparticles. The C moiety constructs a channel for photo-generated electrons between iron oxide andTiO_2_, facilitating the electron transfer from the γ-Fe_2_O_3_ moiety to TiO_2_ moiety. The double-layer structure formed by stacking nanosheets has large pores, which alleviates the transmission of reactants in the catalyst. These constructs also inhibit the separation of electron-hole pairs and achieve a high treatment effect. In this study, phenol was selected as an indicator to study the catalyst’s efficiency for photocatalytic oxidation. We found that two-step charge generation and charge transfer were matched and synergistically enhanced in the non-stacked γ-Fe_2_O_3_/C@TiO_2_ double-layer hollow nanoparticles, which significantly improved the photodegradation of phenol.

## 2. Materials and Methods

### 2.1. Catalyst Preparation

#### 2.1.1. Materials

Ferrocene, hydrogen peroxide (H_2_O_2_), acetone, phenol, BaSO_4_ and ethanol anhydrous were guarantee-grade reagents from Beijing Chemicals Co. Ltd. (Beijing, China). Concentrated ammonia solution (28 wt%), ethylene glycol, sodium acetate, tetra-butyl titanate (TBOT), tetraethyl orthosilicate (TEOS), HCl, NaOH and trisodium citrate were purchased from Tianjin Chemical Corp (Tianjin, China) and were analytical grade. All the water used for all experiments was deionized.

#### 2.1.2. Synthesis of Non-Stacked γ-Fe_2_O_3_/C@TiO_2_ Double-Layer Hollow Nanoparticles

**Figure 1:**
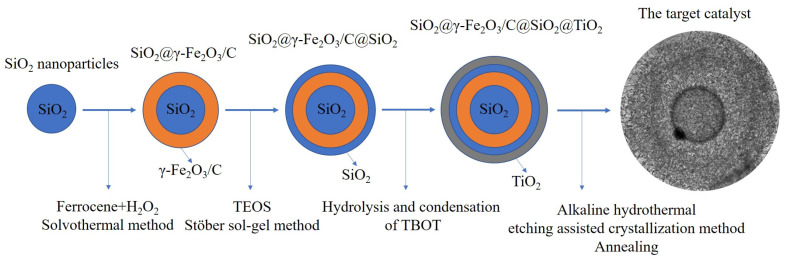
Catalyst synthesis process.

Figure 1 shows the synthesis approaches for the non-stacked γ-Fe_2_O_3_/C@TiO_2_ double-layer hollow nanoparticles. In this fraction, the nanoparticles were prepared by the following steps.

(1) Synthesis of SiO_2_@γ-Fe_2_O_3_/C nanoparticles

Monodispersed SiO_2_ nanoparticles were first synthesized through the Stöber method. A modified hydrothermal approach prepared the core-shell SiO_2_/γ-Fe_2_O_3_/C nanoparticles. Silica nanoparticles (100 mg) and ferrocene (200 mg) were suspended in ethanol (65 mL) using ultrasonic for 30 min. Then, 2 mL H_2_O_2_ was dropwise counted into the mixture and cruised with energetic stirring for 1.5 h. A Teflon-lined stainless-steel autoclave(TEFIC BIOTECH CO., Xi'an, China) was used to heat the homogeneous solution at 210 °C for 48 h. The product was obtained after the autoclave cooled down to room temperature (3 h). Acetone and ethanol were used to wash the obtained products 3 times, respectively, and then the product was vacuum-dried for 12 h.

(2) Synthesis of SiO_2_@γ-Fe_2_O_3_/C@SiO_2_ nanoparticles

The Fe_2_O_3_/C@SiO_2_ nanoparticles (0.3 g) obtained from the last step were added into a three-neck round-bottom flask filled with the ammonia solution (5.0 mL, 28 wt%), ethanol anhydrous (280 mL), and deionized water (70 mL), ultrasonicate for 20 min. TEOS (4 mL) was added dropwise to the mixture in 10 min. The reaction mixture was mechanically stirred for 10 h at room temperature before the products separated. Then the final product was then washed 3 times, respectively, using ethanol and deionized water, and dried in vacuum.

(3) Synthesis of SiO_2_@γ-Fe_2_O_3_/C@SiO_2_@TiO_2_ nanoparticles

The core-shell SiO_2_@γ-Fe_2_O_3_/C@SiO_2_@TiO_2_ nanoparticles were synthesized using the condensation and hydrolysis of TBOT. Ethanol anhydrous (200 mL) was used to disperse the obtained SiO_2_@γ-Fe_2_O_3_/C@SiO_2_ nanoparticles (0.15 g) from the above procedure. Ammonia solution (0.9 mL, 28 wt%) was counted to the mixture and ultrasonicated for 15 min. TBOT (2.0 mL) was then added dropwise to the mixture in 5 min, followed by continuous mechanical stirring for 24 h at 45 °C. The products were separated by centrifugation and washed 3 times, using ethanol and deionized water, and dried under vacuum.

(4) Synthesis of non-stacked γ-Fe_2_O_3_/C@TiO_2_ double-layer hollow nanoparticles

An alkaline hydrothermal etching-assisted crystallization approach was used to synthesize the final products, the SiO_2_@γ-Fe_2_O_3_/C@SiO_2_@TiO_2_ nanoparticles. The SiO_2_@γ-Fe_2_O_3_/C@SiO_2_@TiO_2_ nanoparticles (0.4 g) obtained above were added into a Teflon-lined stainless-steel autoclave (50 mL) filled with a NaOH aqueous solution (25 mL, 2.0 M). The autoclave was sealed and heated to 100 °C for 4 h and cooled to room temperature in 3 h before the next step. The final SiO_2_@γ-Fe_2_O_3_/C@SiO_2_@TiO_2_ nanoparticles were submerged in a HCl aqueous solution (100 mL, 0.1 M) for 20 min followed by washing with deionized water until pH value was close to 7. The final nanoparticles were then dried at 60 °C for 5 h. Finally, the catalyst was annealed at 400 °C for 4 h in an oxygen-deficient environment.

### 2.2. Catalyst Characterization

X-ray powder diffraction (XRD) patterns with 2θ ranging from 10 to 80° (40 kV and 30 mA, D/MAX-2500, Rigaku, Japan), as well as transmission electron microscopy (TEM, JEM-2100 Felectron microscope operating at 200 kV, Tokyo, Japan) were used to obtain the crystal and morphological structure. The samples’ specific surface areas were characterized by the Brunauer–Emmett–Teller (BET) model. Quadrasorb SI analyzer was used to obtain the N_2_ adsorption-desorption isotherms at 77 K. The Barrett–Joyner–Halenda (BJH) model was used to analyze the pore size and volume. X-ray photoelectron spectroscopy (XPS) was performed using a 5300 ESCA equipment (PerkinElmer PHI Co.,Hopkinton, Massachusetts, USA) with an Al Kα X-ray source (250 W) to investigate the chemical compositions of samples. A UV–vis spectrophotometer (UV-2600, Shimadzu, Tokyo, Japan) with BaSO_4_ was used to acquire the UV–vis diffuse reflection spectra (UV–vis DRS).

### 2.3. Catalytic Activity Measurement

The photocatalytic activity was evaluated using a photochemical reactor (TG-10B, Beijing, China) under xenon lamp (300 W). In each experiment, 0.1 g catalyst was weighed in a quartz glass tube, and 30 mL phenol (20 mg L^−1^) was added into it. Before photocatalytic reaction, a dark reaction was carried out for 30 min to achieve the equilibrium of adsorption and desorption of phenol. The absorbency of phenol was determined by a UV-VIS spectrophotometer.

According to absorbance conversion of phenol, the formula of photocatalytic reaction removal rate was shown in (1).

(1) $$D\%=\frac{A_{0}-A_{t}}{A_{0}}\times 100\%$$

*A*_0_—the initial absorbance of phenol.
*A_t_*—Absorption of phenol at t min.

## 3. Results and Discussion

### 3.1. Textural Properties of Catalysts

**Figure 2:**
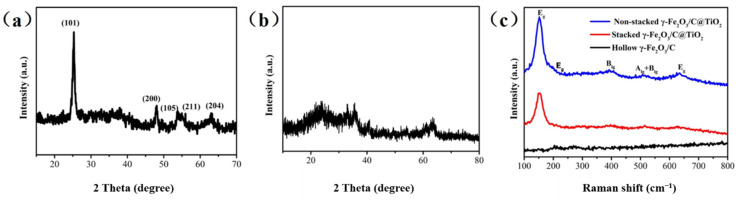
XRD patterns of non-stacked γ-Fe_2_O_3_/C@TiO_2_ (**a**) and hollow γ-Fe_2_O_3_/C (**b**); Raman spectra (**c**) of non-stacked γ-Fe_2_O_3_/C@TiO_2_, stacked γ-Fe_2_O_3_/C@TiO_2_, and hollow γ-Fe_2_O_3_/C.

The wide-angle XRD pattern of non-stacked γ-Fe_2_O_3_/C@TiO_2_ is shown in Figure 2a. The characteristic peaks of the catalyst were indexed to anatase TiO_2_ (JCPDS-ICDD21-1272) [21,22,23]. Other phases such as γ-Fe_2_O_3_ were not observed because the thick TiO_2_ shell passivated the X-ray diffraction. To better analyse the phase composition of the catalyst inner layer, the precursor (hollow γ-Fe_2_O_3_/C) of γ-Fe_2_O_3_/C@TiO_2_ was characterized by XRD also. As shown in Figure 2b, the diffraction peaks of γ-Fe_2_O_3_ (JCPDS-ICDD 25-1402) were observed in hollow γ-Fe_2_O_3_/C [24,25]. Figure 2c shows the Raman spectra of hollow γ-Fe_2_O_3_/C, stacked γ-Fe_2_O_3_/C@TiO_2_, and non-stacked γ-Fe_2_O_3_/C@TiO_2_. The five vibrational modes, located at 640, 545, 395, 193, and 142 cm^−1^, represent E_g_ Raman active, A_1g_ + B_1g_, B_1g_, e.g., and E_g_ modes, indicating that the anatase is the dominant phase of the TiO_2_ hollow spheres.

**Figure 3:**
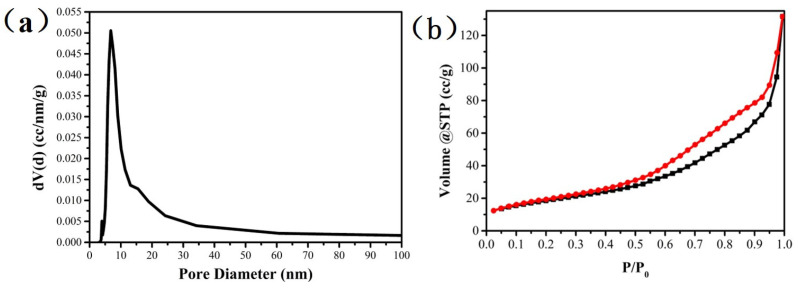
Pore size distribution (**a**) and N_2_ hysteresis curve (**b**) of non-stacked γ-Fe_2_O_3_/C@TiO_2_.

The N_2_ adsorption-desorption was used to measure the specific surface area of the catalyst. Type IV isothermal curves and N_2_ hysteresis loops were observed in Figure 3a, which indicated that the non-stacked γ-Fe_2_O_3_/C@TiO_2_ was a mesoporous material. The pore volume and pore size distributions were also characterized. As shown in Figure 3b, the pore size was mainly distributed from 5–30 nm, which indicated the catalysts’ mesoporous nature. The hollow structure expected to endow the material with a larger specific surface area (145.328 m^2^/g), which has a strong ability to absorb pollution molecules so that the active species produced on its surface can directly react with pollutants and enhance the catalytic performance.

**Figure 4:**
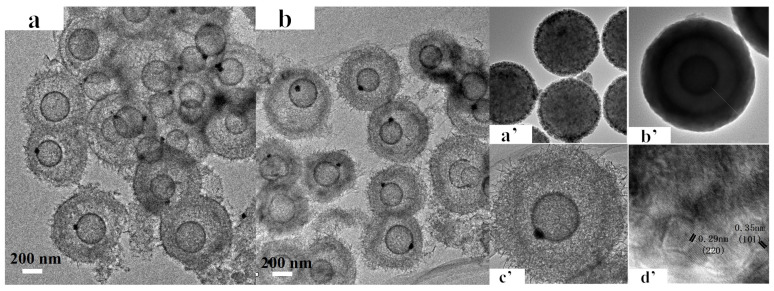
(**a**,**b**) the images of non-stacked γ-Fe_2_O_3_/C@TiO_2_; (**a’**–**c’**) of the process of catalyst preparation, and (**a’**) the image of SiO_2_@γ-Fe_2_O_3_/C; (**b’**) the image of SiO_2_@γ-Fe_2_O_3_/C@SiO_2_@TiO_2_; (**c’**) the image of image of a single sphere of non-stacked γ-Fe_2_O_3_/C@TiO_2_; (**d’**) HRTEM images.

As shown in Figure 4a’, the γ-Fe_2_O_3_/C layer was firmly attached to the outer surface of the SiO_2_ sphere, and the wrapped SiO_2_@γ-Fe_2_O_3_/C nanoparticles exhibited relatively uniform spherical structures. Then, the thin layers of SiO_2_ and TiO_2_ were successfully coated on the surface of the SiO_2_@γ-Fe_2_O_3_/C core to form uniform SiO_2_@γ-Fe_2_O_3_/C@SiO_2_@TiO_2_ nanoparticles (Figure 4b’). After treatment in a hot alkaline solution, the silicon component was removed, and the TiO_2_ expanded both inside and outside to form non-stacked γ-Fe_2_O_3_/C@TiO_2_(Figure 4c’). ICP-AES results showed the nominal ratio of the components (Fe:Ti) was 3.9:1. Furthermore, from Figure 4a,b, almost all catalyst particles can maintain this particular structure. A clear lattice plane was exposed with interplanar spacings of 0.29 nm and 0.35 nm, which revealed the presence of anatase TiO_2_ and γ-Fe_2_O_3_/C, respectively, which mainly expressed the (220) and (101) lattice planes (Figure 4d’).

**Figure 5:**
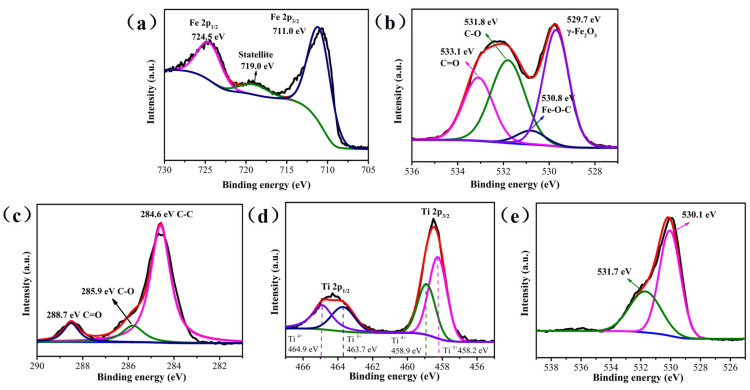
XPS Fe 2p spectrum (**a**), O1s inner shell (**b**), C1s (**c**), Ti2p (**d**), and O1s outer shell (**e**) for non-stacked γ-Fe_2_O_3_/C@TiO_2_.

To further investigate the composition distribution of the inner shell, the Fe, C, and O species located in hollow γ-Fe_2_O_3_/C were characterized by XPS. The typical peaks of Fe2p_3/2_ and Fe2p_1/2_ were located at 711.0 and 724.5 eV, respectively (Figure 5a) [26,27,28]. The presence of the characteristic satellite peak at 719.0 eV confirmed the formation of γ-Fe_2_O_3_, which is the key characteristic distinguishing Fe_2_O_3_ from Fe_3_O_4_. In the O1s spectra (Figure 5b), the presence of Fe-O-C indicated strong interactions between γ-Fe_2_O_3_ and C, which may serve as an electron pathway to facilitate electron transfer via Fe-O-C. Two small peaks at 285.9 and 288.7 eV correspond to the residual C = O and C-O species in the nano-shell after calcination (Figure 5c). The distribution of Ti and O species located in the shell of non-stacked γ-Fe_2_O_3_/C@TiO_2_ is shown in Figure 5d,e. The peaks at binding energies of 464.9 and 458.9 eV were assigned to the Ti2p_1/2_ and Ti2p_3/2_ core levels of Ti^4+^, respectively. The two peaks located at 458.2 and 463.7 eV corresponded to the characteristic peaks of Ti2p_3/2_ and Ti2p_1/2_ of Ti^3+^. The O1s spectra displayed two major oxygen peaks at 530.1 and 531.7 eV, which were attributed to lattice oxygen (O_lat_) and surface-absorbed oxygen (O_sur_), respectively. The lattice oxygen species are nucleophilic reagents that are usually responsible for oxidation reactions.

### 3.2. UV-Vis Absorbance Spectra of Non-Stacked γ-Fe_2_O_3_/C@TiO_2_

**Figure 6:**
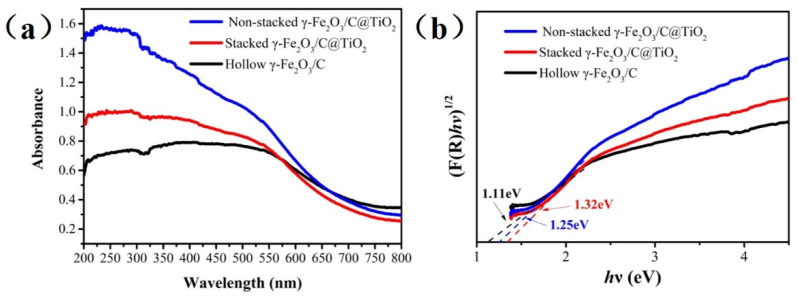
UV-Vis diffuse reflectance spectra (**a**) and calculated band gap patterns based on UV-Vis diffuse reflectance spectra (**b**) of non-stacked γ-Fe_2_O_3_/C@TiO_2_, stacked γ-Fe_2_O_3_/C@TiO_2_, and hollow γ-Fe_2_O_3_/C.

The optical absorption of TiO_2_ was affected by impurities and changes in the bandgap. The impact of catalysts’ overlap modes on the absorption of visible light was analyzed using UV-vis-DRS. Figure 6a shows that the three catalysts generated the absorption band-edge around 550 nm, corresponding to the transition of electrons from the top of the valence band to the footing of the conduction band. The absorption band edge did not immediately decline to 0 from 550 to 800 nm; however, it entered an extended buffer period, corresponding to the energy of photon needed to transition electrons from the O2p level to the impurity station. This extensive absorption range reflected the main characteristics of γ-Fe_2_O_3_/C.

**Figure 7:**
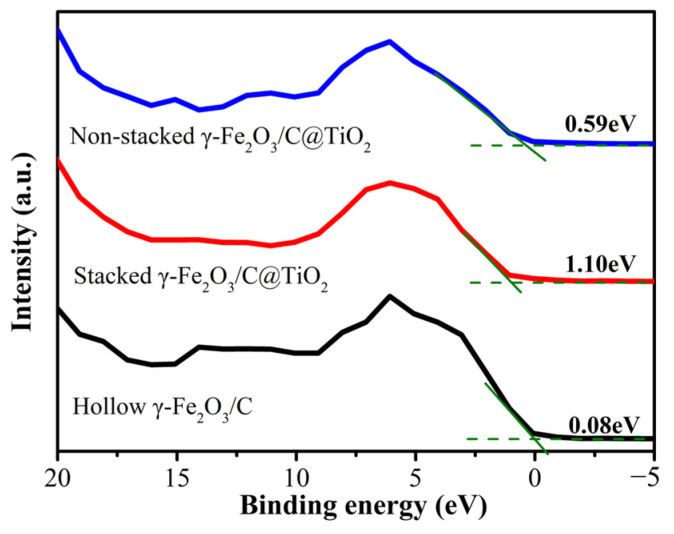
Valance band XPS spectra of non-stacked γ-Fe_2_O_3_/C@TiO_2_, stacked γ-Fe_2_O_3_/C@TiO_2_, and hollow γ-Fe_2_O_3_/C.

From Figure 6b, the first maximum at 2.3 eV (540 nm) is associated with point lattice defects, namely oxygen vacancies, while the subsequent growth at energies above 2.3 eV corresponds to the fundamental bandgap [29,30]. The corresponding bandgap energies of non-stacked γ-Fe_2_O_3_/C@TiO_2_ and stacked γ-Fe_2_O_3_/C@TiO_2_ were 1.25 and 1.32 eV (Figure 6b), which were both higher than hollow γ-Fe_2_O_3_/C (1.11 eV). The band locations (Figure 7) were calculated using the XPS valence spectra and bandgap energies. The γ-Fe_2_O_3_/C exhibited a valence band (VB) at 0.08 eV and a conduction band (CB) at −1.03 eV. The CB and VB of stacked γ-Fe_2_O_3_/C@TiO_2_ and non-stacked γ-Fe_2_O_3_/C@TiO_2_ were notably different. According to previous literature, the shell affects the energy band depending on the material [31,32,33,34]. The TiO_2_ layer had a more positive conduction band voltage than γ-Fe_2_O_3_. The surface dipole layer formed by the TiO_2_ shell caused the conduction band voltage of the γ-Fe_2_O_3_ inner layer to migrate, and the migration direction and magnitude depended on the dipole parameters due to the usage of the two materials and the different stacking modes. Non-stacked γ-Fe_2_O_3_/C@TiO_2_ had a moderate band edge position (VB at 0.59 eV and CB at −0.66 eV). During the photodegradation of organic pollutants, the suitable band edge position enabled three active substances (·OH, ·O^2−^, holes) to function simultaneously.

**Scheme 1:**
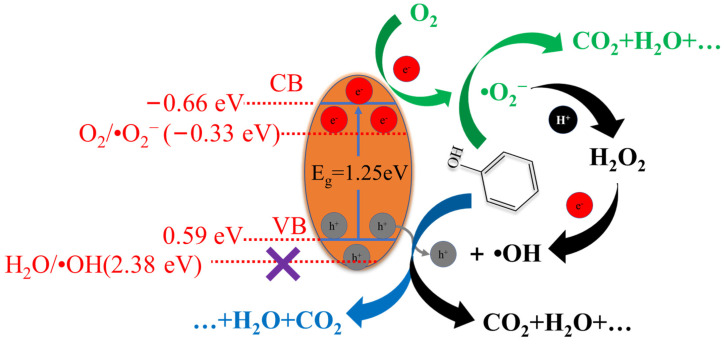
Schematic illustration of the non-stacked γ-Fe_2_O_3_/C@TiO_2_.

The charge generation and charge transfer behaviors over non-stacked γ-Fe_2_O_3_/C@TiO_2_ are proposed in Scheme 1. Incident light can induce the transition of γ-Fe_2_O_3_. Excited electrons generated in γ-Fe_2_O_3_ can be efficiently transmitted to the conduction band of TiO_2_ through C species while holes remain in the valence band of γ-Fe_2_O_3_, resulting in effective electron-hole separation. Therefore, the bandgap will become smaller, and the excitation wavelength range of the TiO_2_ composite was expanded into the visible light region. From Scheme 1, the ·OH, ·O^2−^ and holes will contribute to the degradation of organic matter, and the hydroxyl radicals are not obtained from hole oxidation of water but from the interaction of electrons, hydrogen ions, and superoxide radicals. And the production process of hydroxyl radical is as follows [11]:

e^−^ + O_2_ = ·O_2_^−^

O_2_^−^ + ·O_2_^−^ + 2H^+^ = H_2_O_2_

H_2_O_2_ + e^−^ = ·OH + OH^−^

### 3.3. Photocatalytic Degradation of Phenol

**Figure 8:**
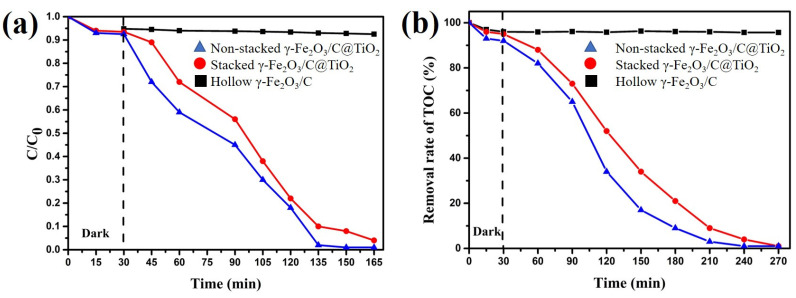
Conversion (**a**) degradation of phenol and (TOC) (**b**) total organic carbon over non-stacked γ-Fe_2_O_3_/C@TiO_2_, stacked γ-Fe_2_O_3_/C@TiO_2_, and hollow γ-Fe_2_O_3_/C catalysts.

Hollow γ-Fe_2_O_3_/C, stacked γ-Fe_2_O_3_/C@TiO_2_, and non-stacked γ-Fe_2_O_3_/C@TiO_2_ photocatalysts were used to degrade phenol under visible light irradiation. As shown in Figure 8a, non-stacked γ-Fe_2_O_3_/C@TiO_2_ showed the highest performance and thoroughly degraded phenol after 135 min of irradiation under Xe lamp with 300 W power (Figure 8b). The catalytic performance was as follows: non-stacked γ-Fe_2_O_3_/C@TiO_2_ > stacked γ-Fe_2_O_3_/C@TiO_2_ > hollow γ-Fe_2_O_3_/C. Compared with stacked γ-Fe_2_O_3_/C@TiO_2_, the performance of non-stacked γ-Fe_2_O_3_/C@TiO_2_ was significantly enhanced due to the unique designed morphology and structure, which can enhance the absorption capacity of visible light and improve the activity of adsorption sites. The hollow inner cavity also permitted the scattering and refraction of light; therefore, the diffusion distance of electron-hole pairs generated by light was reduced, maximizing light utilization. Simultaneously, the unique structure also expanded the specific surface area of the catalytic material. The carbon species can facilitate charge transfer from γ-Fe_2_O_3_ to the outer TiO_2_, reducing the recombination probability of photogenerated carriers. The reusability of non-stacked γ-Fe_2_O_3_/C@TiO_2_ was investigated (Figure S1), and the catalytic activity of the catalyst decreased by only about 5.0% after 5 catalytic cycles.

**Figure 9:**
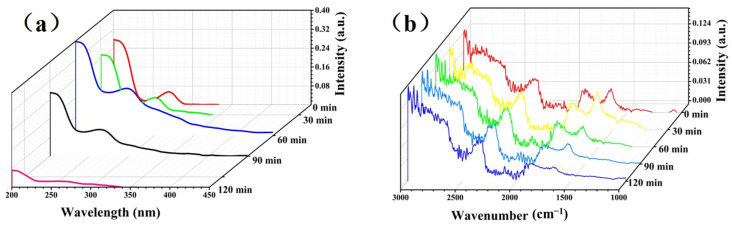
UV-Vis absorbance spectra (**a**), in situ DRIFTS (**b**) of phenol degradation over non-stacked γ-Fe_2_O_3_/C@TiO_2_.

The degradation of phenol using the non-stacked γ-Fe_2_O_3_/C@TiO_2_ was analysed by UV-Vis absorbance spectra (Figure 9a) and in situ DRIFTS (Figure 9b). Interestingly, Figure 9a shows that the absorption band (269 nm) of phenol over non-stacked γ-Fe_2_O_3_/C@TiO_2_ substantially increased upon prolonging the irradiation time beyond 30 min, suggesting the formation of more intermediate products. When the irradiation time was prolonged, the intermediate products formed during the degradation of phenol decomposed to form small molecules, consistent with the results of in situ DRIFTS (Figure 9b). The absorption bands centered at 1410 cm^−1^ represented the stretching vibration of -OH functional group of phenol. The characteristic peaks of phenol significantly decreased when exposed to light after 30 min. This shows that the phenolic -OH was first oxidized during phenol degradation.

**Figure 10:**
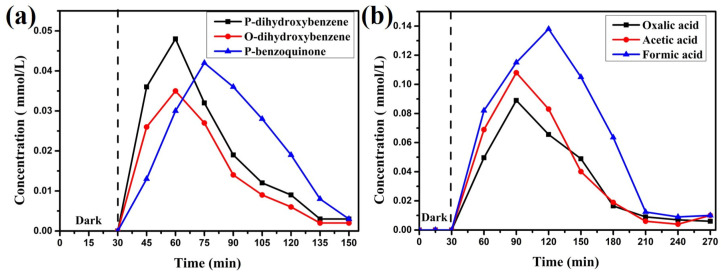
Intermediate products (**a**,**b**) for phenol degradation over non-stacked γ-Fe_2_O_3_/C@TiO_2_.

To further investigate the degradation of phenol over non-stacked γ-Fe_2_O_3_/C@TiO_2_, the intermediate products (*p*-dihydroxybenzene, *o*-dihydroxybenzene, *p*-benzoquinone, oxalic acid, acetic acid, and formic acid) were measured by high-performance liquid chromatography (Figure 10a,b). Under light irradiation, the holes formed by γ-Fe_2_O_3_/C@TiO_2_ reacted with phenol to generate unstable free radicals containing phenoxy groups. The *o*-and *p*-phenolic hydroxyl groups tend to form stable o-dihydroxybenzene, *p*-dihydroxybenzene, which were further oxidized to *p*-benzoquinone. Small molecules, such as oxalic acid, acetic acid, and formic acid, were formed through ring-opening and were eventually mineralized into CO_2_ and H_2_O. Non-stacked γ-Fe_2_O_3_/C@TiO_2_ had a moderate band edge position, allowing it to excite hydroxyl radicals, superoxide free radicals, and holes to oxidize organic compounds. This was ascribed to an appropriate overlap mode between the two layers. Moreover, Table 1 shows a comparison of the double-layer hollow nanoparticles with the other catalysts; it is found that the catalysts in this paper have better catalytic performance and can use fewer catalysts in a short time to achieve higher catalytic efficiency.

## 4. Conclusions

A facile route was used to synthesize non-stacked γ-Fe_2_O_3_/C@TiO_2_ double-layer hollow nanoparticles with appropriate overlap mode between the two layers. This unique material structure reduces the catalyst’s energy band, which can broaden the light response range to the visible light absorption range, and reduces the recombination rate of photogenerated carriers owing to the C moiety facilitated electron transfer from the γ-Fe_2_O_3_ moiety to TiO_2_. The non-stacked γ-Fe_2_O_3_/C@TiO_2_ displayed an excellent photocatalytic performance for phenol degradation under visible light irradiation, which is attributed to the cooperativity of the existence of ·OH, ·O^2-^, holes. In situ DRIFTS further explored the degradation pathway of phenol; during phenol degradation, -OH was first oxidized, and combined with the identification results of intermediate products, then the benzene rings were destroyed to form small molecule organic acid and eventually mineralized into CO_2_ and H_2_O. This overlap mode enhanced both charge generation and charge transfer over photocatalysts. The γ-Fe_2_O_3_ moiety endows the nano-shell with an ability of charge generation, while the C moiety facilitated electron transfer from the γ-Fe_2_O_3_ moiety to TiO_2_. The unique non-stacked double-layer structure inhibited the initial charge recombination in TiO_2_. The non-stacked γ-Fe_2_O_3_/C@TiO_2_ double-layer hollow nanoparticles can make the two steps of charge generation and charge transfer well-matched and synergistically enhanced, which significantly improved the efficiency of the photodegradation of phenol. The preparation of non-stacked γ-Fe_2_O_3_/C@TiO_2_ catalysts also provides a novel approach for the future design of a higher-efficiency photocatalytic system.

## Figures and Tables

**Table 1:** Comparison of catalyst performance.

Photo Catalyst	Phenol	Characteristics	% Degradation
Er^3+^: YAlO_3_/TiO_2_: 0.39 g	50 mgL^−1^	Visible light	58% (8 h) [35]
N-doped TiO_2_: 0.3 g	50 mgL^−1^	λ = 312 nm UV	99.6% (540 min) [36]
2P-TiO_2_-500: 50 mg	10 mgL^−1^	Xenon lamp (300 W)	100% (180 min) [37]
TiO_2_-CdS-gCNNSs: 50 mg	10 mgL^−1^	Visible light	80% (300 min) [38]
TiO_2_/MoS_2_ heterostructures	-	Visible light	78% (150 min) [39]
This work: 0.1 g	20 mgL^−1^	Visible light	Almost 100% (135 min)

## Data Availability

The data presented in this study are available on request from the corresponding author upon reasonable request.

## Supplementary Materials

The following supporting information can be downloaded at: https://www.mdpi.com/article/10.3390/nano12020201/s1, Figure S1: The lifetime of Non-stacked γ-Fe_2_O_3_/C@TiO_2_.
