# Supplementary material for: Non-Stacked γ-Fe2O3/C@TiO2 Double-Layer Hollow Nanoparticles for Enhanced Photocatalytic Applications under Visible Light
Source: Nanomaterials (Basel). 2022 Jan 7;12(2):201. doi: 10.3390/nano12020201 (PMC8779976; doi:10.3390/nano12020201)
Supplement: Supplementary file 1 [file nanomaterials-12-00201-s001.zip › nanomaterials-1502948-supplementary.pdf]

nanomaterials

Supplementary Materials

Non-Stacked γ-Fe_2O_3/C@TiO_2 Double-Layer Hollow Nanoparticles for Enhanced Photocatalytic Applications under Visible Light

Xun Sun [1,*], Xiao Yan [1], Huijuan Su [1], Libo Sun [1], Lijun Zhao [1], Junjie Shi [1], Zifan Wang [2,*], Jianrui Niu [3], Hengli Qian [3] and Erhong Duan [3,*]

[1] Yantai Key Laboratory of Gold Catalysis and Engineering, Shandong Applied Research Center of Gold Nanotechnology (Au-SDARC), School of Chemistry & Chemical Engineering, Yantai University, Yantai 264005, China; yhzheng@ytu.edu.cn (X.Y.); suhuijuan2012@ytu.edu.cn (H.S.); sunlibo@ytu.edu.cn (L.S.); zhaoljytu@ytu.edu.cn (L.Z.); junjieshi@ufl.edu (J.S.)

[2] Biomedical Research Center, Northwest Minzu University, Lanzhou 730000, China

[3] School of Environmental Science and Engineering, Hebei University of Science and Technology, Shijiazhuang 050018, China; niujianrui@hebust.edu.cn (J.N); 211706022@stu.hubest.edu.cn (H.Q)

* Correspondence: sunxun@ytu.edu.cn (X.S.); zifan.wang@mso.umt.edu (Z.W.); deh@hebust.edu.cn (E.D.)

As shown in Figure S1, the catalytic activity of non-stacked γ-Fe_2O_3/C@TiO_2 decreased by only 5.0% during no less than 5 catalytic cycles, which showed enhanced reusability of catalyst applied in degradation of phenol.

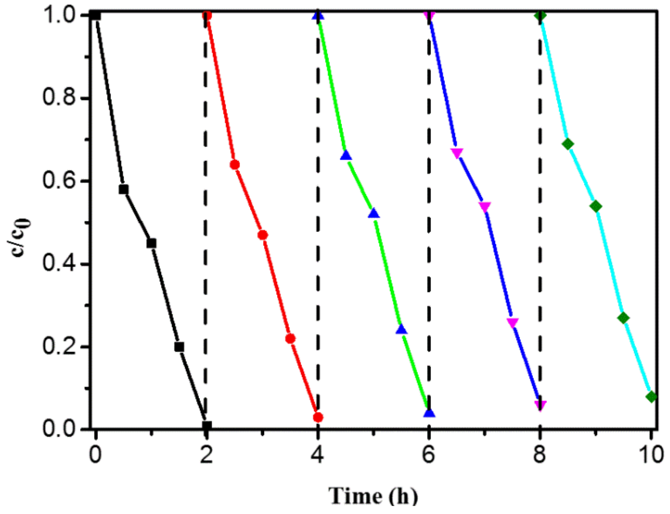

Figure S1. The lifetime of Non-stacked γ-Fe_2O_3/C@TiO_2.
